# Handgrip performance in relation to self-perceived fatigue, physical functioning and circulating IL-6 in elderly persons without inflammation

**DOI:** 10.1186/1471-2318-7-5

**Published:** 2007-03-01

**Authors:** Ivan Bautmans, Ellen Gorus, Rose Njemini, Tony Mets

**Affiliations:** 1 Gerontology, Free University of Brussels (VUB), Laarbeeklaan 103, B-1090 Brussels, Belgium; 2 Rehabilitation Sciences & Physical Therapy, Free University of Brussels (VUB), Laarbeeklaan 103, B-1090 Brussels, Belgium; 3 Geriatrics, University Hospital of the Free University of Brussels (UZ-VUB), Laarbeeklaan 101, B-1090 Brussels, Belgium

## Abstract

**Background:**

Low grip strength is recognized as one of the characteristics of frailty, as are systemic inflammation and the sensation of fatigue. Contrary to maximal grip strength, the physical resistance of the muscles to fatigue is not often included in the clinical evaluation of elderly patients. The aim of this study was to investigate if the grip strength and the resistance of the handgrip muscles to fatigue are related to self-perceived fatigue, physical functioning and circulating IL-6 in independently living elderly persons.

**Methods:**

Forty elderly subjects (15 female and 25 male, mean age 75 ± 5 years) were assessed for maximal grip strength, as well as for fatigue resistance and grip work (respectively time and work delivered until grip strength drops to 50% of its maximum during sustained contraction), self perceived fatigue (VAS-Fatigue, Mob-Tiredness scale and the energy & fatigue items of the WHOQOL-100), self rated physical functioning (domain of physical functioning on the MOS short-form) and circulating IL-6. Relationships between handgrip performance and the other outcome measures were assessed.

**Results:**

In the male participants, fatigue resistance was negatively related to actual sensation of fatigue (VAS-F, p < .05) and positively to circulating IL-6 (p < .05). When corrected for body weight, the relations of fatigue resistance with self-perceived fatigue became stronger and also apparent in the female. Grip strength and grip work were significantly related with several items of self-perceived fatigue and with physical functioning. These relations became more visible by means of higher correlation coefficients when grip strength and grip work were corrected for body weight.

**Conclusion:**

Well functioning elderly subjects presenting less handmuscle fatigue resistance and weaker grip strength are more fatigued, experience more tiredness during daily activities and are more bothered by fatigue sensations. Body weight seems to play an important role in the relation of muscle performance to fatigue perception. Elderly patients complaining from fatigue should be physically assessed, both evaluating maximal grip strength and fatigue resistance, allowing the calculation of grip work, which integrates both parameters. Grip work might best reflect the functional capacity resulting from the development of a certain strength level in relation to the time it can be maintained.

## Background

With advancing age skeletal muscle mass and strength decrease dramatically, a phenomenon called sarcopenia [1]. In aging research, grip strength has often been used as an indicator of general muscle strength, since it is a parameter easy to measure. Indeed, age-related changes in grip strength are well described [2] and appear to be similar to the strength losses in other muscle groups [3]. Therefore, grip strength is a useful tool in the clinical evaluation of geriatric patients [3,4]. Severe inflammation, as seen during acute infections, dramatically worsens sarcopenia-induced reduction in grip strength [5]. Also, in well-functioning elderly persons chronic low-grade inflammation is associated with a worse degree of sarcopenia and reduced grip strength [6]. Low grip strength is recognized as one of the characteristics of frailty, as are inflammation and the sensation of fatigue [4,7-10]. Due to sarcopenia, older or ill persons will function closer to their limit of maximal strength [11,12]. Since daily activities in the elderly often require sustained intense muscle contractions (e.g. when bearing shopping bags), these may be more challenging given the reduced muscle strength, and could explain the common sensation of fatigue. This tiredness during daily activities is not a trivial symptom but a predictor of disability and mortality in older individuals [13,14].

In a general population, the sensation of fatigue is very common (in respectively 20.4% and 14.3% of women and men) [15]; it is predominantly (in 98%) present in residents of long-term care facilities [16]. Contrary to maximal grip strength, the physical resistance of the muscles to fatigue is not often measured in the clinical evaluation of elderly patients. Previously, we have studied grip strength as well as handgrip fatigue resistance in elderly subjects in different clinical conditions [5,17,18]. Subjects were instructed to sustain maximal grip effort as long as possible, and fatigue resistance was expressed as the time during which grip strength dropped to 50% of its maximum. From these studies, the handgrip fatigue resistance, more than grip strength, appeared to be closely related to the clinical condition of the subjects. However, it is not clear whether reduced muscle fatigue resistance is also related to sensations of fatigue.

The aim of this study was to investigate if the physical resistance of the handgrip muscles to fatigue is related to self-perceived fatigue and physical functioning in elderly community-dwelling persons. Since inflammation is related to both reduced muscle fatigue resistance [5,18] and increased fatigue perception [19,20], here we report a first explorative study in elderly persons without inflammation. In this study, we describe a parameter reflecting the work delivered by the muscles of the forearm during the fatigue resistance test, which we called Grip Work. In fact, muscle strength becomes functional when it can be maintained for a certain time. Also, resistance to fatigue of the muscles is only relevant in daily life when sufficient muscle strength can be developed. The capacity to generate a certain amount of muscle work attains functionally significant dimensions when it can be applied to deliver mechanical work, i.e. to move a given weight over a certain distance. In this context, the body weight of the subject can be of critical importance and heavier subjects might encounter more difficulty and might experience more fatigue when functioning with similar muscle performance than leaner persons. Therefore, we have also corrected the scores of handgrip performance for body weight. Finally, the relation of handgrip performance with circulating Interleukin(IL)-6 was investigated. Previously, we found negative correlations between IL-6 with grip strength and fatigue resistance in inflammatory geriatric patients [5,18]. Elderly, even when considered heallthy, frequently present low-grade inflammation [21]. Also, IL-6, besides its implication in acute inflammation, has been reported to have a signalling function, linked to muscle performance [22].

## Methods

### Participants

Two hundred and seventy four elderly (144 female and 130 male, mean age 76.4 ± 5.4 years) enrolled in a longitudinal survey conducted by our department and living independently in and around Brussels (Belgium), were invited by letter to participate in the study. All subjects were recently screened for Alzheimer's disease and presented a normal cognitive function (mean Mini Mental State Examination score 27.9 ± 1.5, MMSE [23]). Inclusion criteria for participation in the study were: male or female, aged 60 years or over, presenting normal cognitive function (MMSE > 23) and living independently in the community. Subjects were excluded if they reported acute or chronic inflammatory illness, used non-steroidal anti-inflammatory drugs or corticoids, or presented elevated circulating C-reactive protein (CRP, >3 mg/L according to the CDC & AHA recommendations [24]). Eighteen female and 27 male volunteers matched the inclusion criteria. Five subjects (two male and three female) presented elevated circulating CRP levels and were excluded. Finally, 15 female and 25 male participated in the study (see table 1). The local ethical committee approved the study protocol and all participants gave an informed consent.

### Measurements

#### Measurement sequence

For each participant all evaluations were performed in the morning of the same day. First, the subjects' height and weight (standing undressed on an analogue weighing scale, Seca, Hamburg, Germany) were measured and from the non-dominant arm blood was drawn for determination of circulating CRP and IL-6. Next, they completed a questionnaire concerning self-perceived fatigue and general health condition. Afterwards, handgrip performance was measured in all participants by the same investigator (IB), who was unaware of the questionnaire data.

#### CRP and IL-6 assay

CRP was determined by nephelometry (Behring, Marburg, Germany; normal value <3 mg/L). Sera were assayed for IL-6 by ELISA (Biosource international, Nijvel, Belgium), according to the manufacturer's instructions. IL-6 concentrations were detected by comparing sample absorbance with the absorbance of a reference purified recombinant cytokine. All determinations were done within one single assay. Intra-assay coefficient of variance (CV) was determined by the manufacturer: CV = 7.7% for low, CV = 5.7% for normal and CV = 5.1% for high standards.

#### Self-perceived fatigue

Three different instruments were used to measure the sensation of fatigue in our participants. Subjects scored their current fatigue-level on a Visual Analogue Scale for Fatigue (VAS-F). Fatigue following daily-life activities was estimated using the Mobility-Tiredness scale (Mob-T), specifically tailored to measure tiredness during daily activities in elderly persons (high scores indicate less fatigue) [25]. The items covering energy and fatigue (WHOQOL-F2.2: "How easily do you get tired?", WHOQOL-F2.4: "How much are you bothered by fatigue?") from the World Health Organization Quality of Life Questionnaire were scored on a 1-to-5 scale going from 'not at all' to 'extremely' [26].

#### General health condition

Co-morbidity and medication use were recorded using self-reporting questionnaires. According to the participant's native language, the French [27] or Dutch [28] version of the Medical Outcome Study (MOS) Short-form was used to estimate self-perceived health; the domain of physical functioning was recorded and transformed to a 0–100 scale [29], with high scores reflecting better functioning.

#### Handgrip performance

Maximal grip strength and fatigue resistance were measured using the Martin vigorimeter (Elmed Inc., Addisson, USA) as described previously [17]. Briefly, the shoulder was adducted and neutrally rotated, elbow flexed at 90°, forearm in neutral position and wrist in slight extension (0 to 30°). Then, the subject was asked to squeeze the large bulb of the vigorimeter as hard as possible. For each hand, the highest of three attempts was noted as the maximal grip strength (in KPa). Afterwards, the subject was instructed to squeeze again the bulb of the vigorimeter as hard as possible and to maintain this maximal pressure; the time (in seconds) during which grip strength dropped to 50% of its maximum was recorded as fatigue resistance. This fatigue resistance test is highly reproducible in elderly subjects with ICC-values ranging respectively from 0.91 to 0.94 and from 0.88 to 0.91 for intra- and inter-observer reliability [17]. An estimate of the total effort produced during the fatigue resistance test, defined as Grip Work, was calculated as

Grip Work = (Grip Strength × 0.75) × Fatigue Resistance

**Figure 1:**
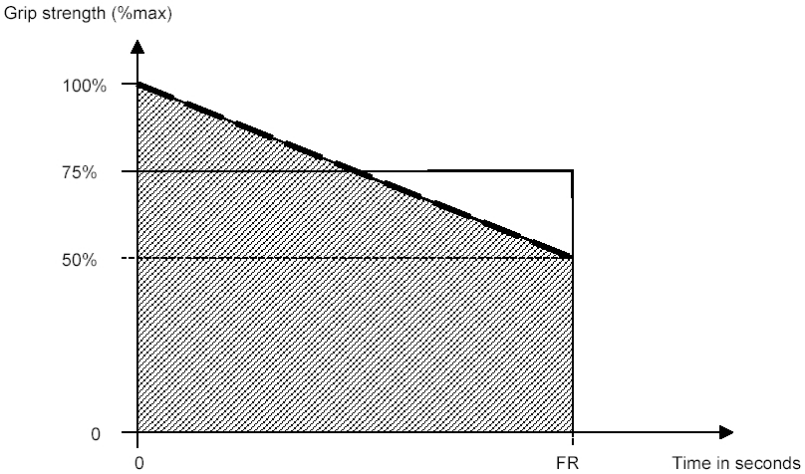
**Schematic presentation of Grip Work**. FR = Fatigue resistance: the time during which grip strength decreases to 50% of its maximum value. The bold line represents the estimate of the evolution of grip strength with time during sustained contraction. Grip work is calculated as FR multiplied by 75% of the maximal grip strength, corresponding to the area under the curve (shaded).

This parameter represents the physiologic work delivered by the handgrip muscles during the fatigue resistance test. When graphically represented, grip work is the area under the curve with grip strength in the vertical and time in the horizontal axis (Figure 1). All handgrip performance tests were executed with the dominant and non-dominant hand, and the mean of both hands was used as final outcome measure.

### Statistical analysis

Statistical analysis was performed using SPSS release 12.0 (SPSS Inc., Chicago, IL USA). Average values are given ± standard deviation (SD). Outcome parameters for fatigue showed either non-normal distribution as evaluated using the Kolmogorov-Smirnov Goodness of Fit Test (fatigue resistance p = .024, fatigue resistance/kg body weight p = .004, VAS-Fatigue p = .004 and Physical Functioning p = .004) or were scored on an ordinal scale (0–6 for Mob-Tiredness scale and 1–5 for WHOQOL F2.2 & F2.4). Therefore, non-parametric tests were used [30]. Since gender differences might interfere with the evaluation of relationships between outcome parameters, differences between male and female participants were explored with the Mann-Whitney U-test prior to compute correlations. Spearman's rho correlation coefficient was computed to investigate relationships between handgrip performance and self-perceived fatigue & physical functioning, and circulating IL-6. Handgrip performance was also expressed per kg body mass and correlations with self-perceived fatigue & physical functioning, and circulating IL-6 were computed. Significance was set a priori at two-sided p < .05.

## Results

First, gender differences on the outcome parameters were assessed. As can be seen in table 2, the male participants were heavier, taller and stronger (all p < .01), and had higher BMI (p < .05) and circulating levels of IL-6 (p < .01) compared to the female. On the other hand, female participants scored better on the fatigue resistance test (p < .01) and had slightly but significantly higher scores on the WHOQOL F2.4 (p < .05).

Given these gender contrasts, relationships between handgrip performance and the other outcome parameters were primarily analysed for the male and female participants separately. However, for illustration purpose, also the correlation coefficients for all participants together are presented (see table 3 and 4). Muscle fatigue resistance in the male participants was negatively correlated to the score on the VAS-F (r = -0.41, p < .05) and positively with circulating levels of IL-6 (r = 0.44, p < .05). No other significant correlations were found between fatigue resistance and self-perceived fatigue & physical function or IL-6, neither in both genders together or separately. In the male subjects, better grip strength was related with less fatigue (all p < .05, except for the item WHOQOL F2.2, see table 3). In both male and female, grip strength and grip work were positively related to self-rated physical functioning (p < .05). Also, better grip work was related to less perceived fatigue (for the male with VAS-F p < .01 and the item WHOQOL F2.4 p < .05; for the female with Mob-T and the item WHOQOL F2.4 both p < .05). In the male subjects, better grip work was related to higher levels of circulating IL-6 (r = 0.42, p < .05).

When corrected for body weight, higher muscle fatigue resistance was significantly related with less perceived fatigue (p < .05 for both male r = -0.45 and female r = -0.63 on the item WHOQOL F2.4, r = -0.42 p < .05 for the male on the VAS-F and r = 0.59 p < .05 for the female on the Mob-T). As shown in table 4, grip strength in the male participants expressed per kg body weight was significantly related to all self-perceived fatigue items (all p < .05, p < .01 on WHOQOL F2.4) as well as with self-rated physical functioning (p < .01). Also in the female, grip strength corrected for body weight related significantly with self-assessed physical functioning (p < .05). In both male and female participants grip work per kg body weight correlated significantly with self-perceived fatigue (for the male p < .01 on VAS-F and on WHOQOL F2.4, and p < .05 for Mob-T; for the female p < .01 on Mob-T and on WHOQOL F2.4). Corrected grip work related positively with self-rated physical functioning (p < .01 in both male and female).

## Discussion

In this explorative study we have investigated the relationships between handgrip performance and the self-perceived sensation of fatigue in well-functioning elderly persons. In elderly subjects, fatigue might not allways be perceived as such and might be expressed as symptoms related to the occurrence of fatigue. Therefore, we have approached fatigue using different instruments, each representing fatigue in another context. On the one hand, actual fatigue sensation was scored as such on a VAS-scale. On the other hand, we evaluated the extend to which fatigue had an impact on the quality of life (WHOQOL F2.2 & F2.4) or was provoked by daily activities (Mob-Tiredness scale). Finally, self-rated physical functioning was recorded, since limitation in physical functioning might also be an expression of fatigue.

In the male subjects muscle fatigue resistance (time until grip strength drops to 50% of its maximum during sustained contraction) was significantly related with the actual sensation of fatigue, as assessed with the VAS-F (p < .05). Also, better grip strength was significantly related to less actual fatigue sensation (VAS-F, p < .05), less tiredness during daily activities (Mob-T, p < .05) and less perturbation due to fatigue (WHOQOL F2.4, p < .05). These findings support the hypothesis that elderly having more muscle strength probably need to engage less of their maximal strength in order to perform daily activities, and thus get less easily fatigued. For the female participants, the correlation coefficients were in the same direction, but did not reach significancy. The way fatigue is perceived might differ between elderly male and femal subjects. In this context, also Wijeratne et al. [31] found some differences in the relationship between musculoskeletal and fatigue symptoms according to gender in elderly persons. Probably, in healthy subjects fatigue resistance reflects mainly the physiological ability of skeletal muscles to sustain maximal contractions, and is less linked to fatigue perception or motivation. Other reports also indicate that during sustained maximal muscle contraction, force drops to 50% of the initial value within about one minute and that almost all loss in force is due to mechanisms within the contracting muscles [32-34].

When analyzing grip work, a parameter reflecting the total work delivered by the handgrip muscles during the fatigue resistance test, significant relations were found with self-rated fatigue in both the male (VAS-F and WHOQOL F2.4, both p < .01) and the female (Mob-T and WHOQOL F2.4, both p < .05). This is the first description of grip work as a parameter for muscle performance. Grip work integrates the maximal muscle strength with the time during which the muscle contraction can be maintained before the strength drops to 50% of its maximum. In fact, maximal grip strength reflects the capacity of the muscles to generate a large force in a very short time. In addition, grip work estimates the ability to sustain that strength in time. Specifically the latter aspect of muscle performance is important during daily activities that need sustained muscle activity (e.g. when lifting, manipulating or bearing objects). The significant relationship of grip work with self-perceived sensation of fatigue supports the validity of this parameter as a functional outcome measure in the clinical evaluation of elderly subjects.

From a functional viewpoint, the generated force output of muscles is efficient when it allows performing daily activities in a comfortable manner. In this context body mass can be of critical importance. In our study, we have corrected the parameters of handgrip performance for body weight and explored their relationships with self-perceived fatigue. When expressed per kg body weight, the muscle fatigue resistance was significantly related to self-rated fatigue (VAS-F for the male and Mob-T for the female, both p < .05) and the extend to which the subjects were bothered by fatigue (WHOQOL F2.4, p < .05 for both male and female). When expressed per kg body weight, also the relations of grip strength and grip work with self perceived fatigue became more visible by means of higher correlation coefficients in both male and female participants. Indeed, daily activities might be more challenging for obese persons compared to leaner subjects having the same muscle performance, and might thus be more easily accompanied with fatigue.

We have also studied the relation of handgrip muscle performance with self-rated physical functioning. Grip strength and grip work were significantly related with physical functioning in both male and female participants. When corrected for body weight, these correlations remained of the same magnitude and statistically significant. However, no significant relationship was found between muscle fatigue resistance and self-rated physical functioning. This might be explained by the fact that fatigue resistance, defined as the ability to maintain one's maximal effort, is independent of the initially produced force. Persons presenting a higher grip strength thus can have the same fatigue resistance as weaker ones. For physical functioning, both the maximal strength as well as the time it can be maintained are of critical importance, and thus the grip work (eventually corrected for body weight) might be the best parameter to measure in the physical evaluation of elderly subjects.

We looked for relationships between handgrip performance and circulating levels of IL-6. In our male participants fatigue resistance and grip work correlated positively (p < .05) with IL-6. This is in contrast with our previous studies in hospitalised geriatric patients, where worse muscle fatigue resistance was significantly related to higher levels of circulating IL-6 [5,18]. Probably the relation of the cytokine IL-6 with muscle performance depends on the situation in which it is released in the blood circulation. In the present study, participants showed no evident inflammatory activity (CRP < 3 mg/L), and thus the signalling role of IL-6 might be different compared to situations accompanied with severe inflammation (CRP > 10 mg/L) as in our previous work [5,18]. Indeed, some authors have pointed out possible mechanisms responsible for positive relations between IL-6 signalling and muscle performance [35,36]. Noteworthy, these relationships were attenuated when handgrip fatigue resistance and grip work were corrected for body weight. It can not be excluded that the correlations with IL-6 were influenced by the body weight of the participants, since it has been shown that adiposity and type-2 diabetes is positively linked to higher levels of circulating IL-6 [37]. However, in that case, lower muscle performance would be expected. Clearly, this 'IL-6 paradox' on muscle performance needs further study.

As a final point, it can not be excluded that some of the non-significant correlations in our study are due to a type-2 error. However, for this study we have contacted 274 elderly subjects with normal cognitive function, from whom a large number had to be excluded. Indeed, given the burden of chronic late-life disease and polypharmacy, many elderly subjects show inflammation or use anti-inflammatory drugs. Although participants included in this study and non-participating elderly were similar regarding age and cognitive function, selection bias cannot be completely excluded. On the other hand, the fact that the study population consisted in completely independent and well functioning elderly persons probably resulted in a low variability of fatigue sensations that might have masked some relationships. It is our opinion that the results of this study support the validity of handgrip fatigue resistance, grip work and maximal grip strength as relevant outcome measures in the physical evaluation of elderly patients. Further research with these evaluation instruments, documenting elderly persons in different clinical settings might contribute to its validity and clinical usefulness.

## Conclusion

From the results of our study we can conclude that handgrip performance is related to self-perceived fatigue and physical function in elderly, independently living persons without inflammation. Weaker and less fatigue resistant elderly subjects are more fatigued, experience more tiredness during daily activities and are more bothered by fatigue sensations. Body weight seems to play an important role in the relation of muscle performance to fatigue perception. Elderly patients complaining from fatigue should be physically assessed, both evaluating maximal grip strength and fatigue resistance, allowing the calculation of grip work, which integrates both parameters. Grip work might best reflect the functional capacity resulting from the development of a certain strength level in relation to the time it can be maintained.

## Competing interests

The author(s) declare that they have no competing interests.

## Authors' contributions

TM and IB conceived the study. TM participated in the coordination of the study, the analysis and the redaction. IB performed the statistical analysis and the redaction, and participated in the coordination of the study and the measurements. EG and RN participated in the recruitment of participants and the measurements. All authors read and approved the final manuscript.

## Pre-publication history

The pre-publication history for this paper can be accessed here:



## Figures and Tables

**Table 1:** Overview of subjects invited to participate.

	**invited to participate (N = 274)**	**matching inclusion criteria (N = 45)**	**Excluded (N = 5)**	**participating in the study (N = 40)**
**Male/Female **(N)	130/144	27/18	2/3	25/15
**Age **(years)	76.4 ± 5.4	75.8 ± 5.5	77.6 ± 6.5	75.5 ± 5.5
**MMSE **(score/30)	27.8 ± 1.5	27.8 ± 1.3	27.0 ± 0.8	28.0 ± 1.4

Values represent number or mean ± Standard Deviation, MMSE = Mini Mental State Evaluation.

**Table 2:** Participants' characteristics.

	**Female **N = 15	**Male **N = 25
**Age **(years)	75.3 ± 6.0	75.6 ± 5.2
**MMSE **(score/30)	27.8 ± 1.7	28.0 ± 1.2
**Weight **(kg)^†^	63.6 ± 10.5	80,6 ± 9.4
**Height **(m)^†^	1.61 ± .06	1.70 ± .05
**Body Mass Index **(kg/m^2^)*	24.5 ± 3.9	28.0 ± 3.1
**Comorbidity **(number)	1.1 ± .7	1.5 ± 1.2
**Medication use **(number)	2.4 ± 1.5	2.8 ± 2.5
**IL-6 **(pg/L)^†^	3,3 ± 1,1	4,9 ± 1,8
**Fatigue Resistance **(sec)^†^	82.3 ± 24.1	54.0 ± 18.8
**Grip Strength **(KPa)^†^	54.4 ± 13.0	73.0 ± 17.2
**Grip Work **(KPa × sec)	3340.6 ± 1224.6	2934.3 ± 1043.7
**VAS-F **(score/100)	33.7 ± 22.9	25.5 ± 24.8
**Mob-Tiredness scale **(score/6)	5.1 ± 1.4	4.8 ± 1.7
**WHOQOL F2.2 **(score/5)	2.7 ± 1.0	2.3 ± .8
**WHOQOL F2.4 **(score/5)*	2.8 ± 1.2	2.0 ± .9
**Physical Functioning **(score/100)	64.5 ± 33.9	70.0 ± 32.3

Values represent number or mean ± Standard Deviation, MMSE = Mini Mental State Evaluation, IL-6 = circulating Interleukin-6, VAS-F = Visual Analogue Scale for Fatigue, WHOQOL = WHO Quality of Life Questionnaire (F2.2 = "How easily do you get tired?", F2.4 = "How much are you bothered by fatigue?", both scored on a 1-to-5 scale going from 'not at all' to 'extremely'). Gender difference *p < .05, ^†^p < .01

**Table 3:** Relationships between Handgrip Performance, Self-Perceived Fatigue and Physical Functioning.

	**Grip Strength**			**Grip Work**		
	*Male*	*Female*	*All*	*Male*	*Female*	*All*

**VAS-F**	-.40*	-.23	-.40*	**-.59**^†^	-.36	-.39*
**Mob-Tiredness scale**	.48*	.36	.40*	.37	.52*	**.42**^†^
**WHOQOL F2.2**	-.33	-.25	-.39*	-.32	-.21	-.24
**WHOQOL F2.4**	-.47*	-.32	**-.53**^†^	**-.51**^†^	-.60*	**-.44**^†^
**Physical Functioning**	**.68**^†^	.57*	**.62**^†^	.50*	.59*	**.57**^†^

Values represent Spearman's Rho Coefficients; *p < .05, ^†^p < .01, VAS-F = Visual Analogue Scale for Fatigue, WHOQOL = WHO Quality of Life Questionnaire (F2.2 = "How easily do you get tired?", F2.4 = "How much are you bothered by fatigue?", both scored on a 1-to-5 scale going from 'not at all' to 'extremely').

**Table 4:** Relationships between Handgrip Performance corrected for Body Weight, Self-Perceived Fatigue and Physical Functioning.

	**Grip Strength/kg body weight**			**Grip Work/kg body weight**		
	*Male*	*Female*	*All*	*Male*	*Female*	*All*

**VAS-Fatigue**	-.41*	-.31	-.36*	**-.55**^†^	-.50	-.35*
**Mob-Tiredness scale**	.49*	.43	**.48**^†^	.42*	**.72**^†^	**.48**^†^
**WHOQOL F2.2**	-.46*	-.37	**-.44**^†^	-.38	-.32	-.24
**WHOQOL F2.4**	**-.57**^†^	-.50	**-.55**^†^	**-.57**^†^	**-.79**^†^	**-.40**^†^
**Physical Functioning**	**.67**^†^	.57*	**.65**^†^	**.53**^†^	**.68**^†^	**.51**^†^

Values represent Spearman's Rho Coefficients; *p < .05, ^†^p < .01, VAS-F = Visual Analogue Scale for Fatigue, WHOQOL = WHO Quality of Life Questionnaire (F2.2 = "How easily do you get tired?", F2.4 = "How much are you bothered by fatigue?", both scored on a 1-to-5 scale going from 'not at all' to 'extremely').
